# Demonstration of neutron time-of-flight diffraction with an event-mode imaging detector

**DOI:** 10.1107/S1600576724004448

**Published:** 2024-07-11

**Authors:** Tim T. Jäger, Adrian S. Losko, Alexander Wolfertz, Søren Schmidt, Mads Bertelsen, Anton Khaplanov, Sean R. Agnew, Fumiaki Funama, Manuel Morgano, Markus Roth, Jason R. Gochanour, Alexander M. Long, Luca Lutterotti, Sven C. Vogel

**Affiliations:** a Material Science and Technology Division, Los Alamos National Laboratory, Los Alamos, NM 87545, USA; b Institut für Kernphysik TU Darmstadt Schlossgartenstrasse 9 64289Darmstadt Germany; c Forschungs-Neutronenquelle Heinz Maier-Leibnitz TU München Lichtenbergstrasse 1 85748Garching Germany; d European Spallation Source ERIC, Data Management and Software Centre, Asmussens Allé 305, 2800 Lyngby, Denmark; e Second Target Station Oak Ridge National Laboratory 1 Bethel Valley Road Oak Ridge TN37830 USA; f Materials Science & Engineering University of Virginia 395 McCormick Road Charlottesville VA22904 USA; g Neutron Optics and Polarization Group Oak Ridge National Laboratory 1 Bethel Valley Road Oak Ridge TN37830 USA; h European Spallation Source ERIC PO Box 176 SE-22100Lund Sweden; i Physics Division, Los Alamos National Laboratory, Los Alamos, NM87545, USA; j Department of Industrial Engineering University of Trento 38123Trento Italy; Australian Nuclear Science and Technology Organisation, Lucas Heights, Australia

**Keywords:** neutron diffraction, detector design, imaging detector

## Abstract

Neutron diffraction beamlines have traditionally relied on deploying large detector arrays of ^3^He tubes or neutron-sensitive scintillators coupled with photomultipliers to efficiently probe crystallographic and microstructure information of a given material. Given the large upfront cost of custom-made data acquisition systems and the recent scarcity of ^3^He, new diffraction beamlines or upgrades to existing ones demand innovative approaches. This paper introduces a novel Timepix3-based event-mode imaging neutron diffraction detector system as well as first results of a silicon powder diffraction measurement made at the HIPPO neutron powder diffractometer at the Los Alamos Neutron Science Center. Notably, these initial measurements were conducted simultaneously with the ^3^He array on HIPPO, enabling direct comparison. Data reduction for this type of data was implemented in the *MAUD* code, enabling Rietveld analysis. Results from the Timepix3-based setup and HIPPO were benchmarked against *McStas* simulations, showing good agreement for peak resolution. With further development, systems such as the one presented here may substantially reduce the cost of detector systems for new neutron instrumentation as well as for upgrades of existing beamlines.

## Abstract 2

This paper demonstrates an event-mode imaging based neutron diffraction detector system that employs a scintillator screen, an image intensifier and a Timepix3-based camera. This highly configurable approach allows for large-solid-angle low-cost neutron diffraction setups with a performance comparable to that of the ^3^He detectors on a time-of-flight neutron diffraction beamline.

## 1. Introduction

Neutron scattering is a widely used technique for investigating the crystal structure (*e.g.* lattice parameters and atom positions) and microstructure (*e.g.* texture, defects, phase fractions) of materials. Covering large areas with neutron detectors is key to efficient scattering experiments (Diawara, 2023 ▸). Most modern neutron scattering instruments rely on ^3^He gas, ^10^B or ^6^Li doped scintillators as the neutron-active component (Kirstein *et al.*, 2014 ▸; Stefanescu *et al.*, 2017 ▸; Diawara, 2023 ▸). Because of the scarcity of ^3^He (Zeitelhack, 2012 ▸) and the number of detector and readout modules required by those systems, the neutron detectors can become a major cost factor for scattering beamlines for new projects as well as upgrades of existing ones. Thus an active search for suitable alternatives is ongoing (Kirstein *et al.*, 2014 ▸; Pietropaolo *et al.*, 2020 ▸), *e.g.* for planned diffraction beamlines such as PIONEER (Liu *et al.*, 2022 ▸) at the Spallation Neutron Source or BEER (Fenske *et al.*, 2016 ▸) at the European Spallation Source.

Recently, Losko *et al.* (2021 ▸) demonstrated the use of a Timepix3-based event-mode imaging camera to detect individual photons emitted from a scintillator screen, and its advantages in cold and thermal neutron imaging. In this previous work, it was established that superior spatial and temporal resolution can be achieved through careful analysis of spatial and temporal photon distributions emitted from a scintillator screen (event clustering). Moreover, background from scintillator afterglow and incident gammas can be subtracted by particle discrimination based on simple scintillation photon multiplicity thresholds and temporal photon distributions. One promising advantage of this system is its use of optics for scintillation photon detection, which allows for adjustable fields of view that can be tailored for any given experimental setup, such as neutron diffraction detectors which require large-area fields of view. Additionally, the entire detector is read out by one chip and one software, avoiding the necessity for thousands of photomultipliers and acquisition channels. While this approach was originally developed and demonstrated for neutron imaging, here we present a first demonstration of the event-mode technique for neutron scattering data acquisition, in particular neutron time-of-flight diffraction, including the adaptation of the *MAUD* (*Materials Analysis Using Diffraction*) Rietveld code (Lutterotti *et al.*, 1999 ▸) to refine the required calibration parameters during the Rietveld analysis.

To compare this novel diffraction detection method utilizing imaging detectors with a conventional neutron time-of-flight diffraction detector system, a silicon powder diffraction experiment was conducted on the High Pressure–Preferred Orientation (HIPPO) beamline (Wenk *et al.*, 2003 ▸; Vogel *et al.*, 2004 ▸; Takajo & Vogel, 2018 ▸) at the Los Alamos Neutron Science Center (Lisowski & Schoenberg, 2006 ▸) Spallation Neutron Source, and simulations with the Monte Carlo code *McStas* (Lefmann & Nielsen, 1999 ▸) were carried out.

## 2. Methods

### 2.1. Experimental setup

**Figure 1:**
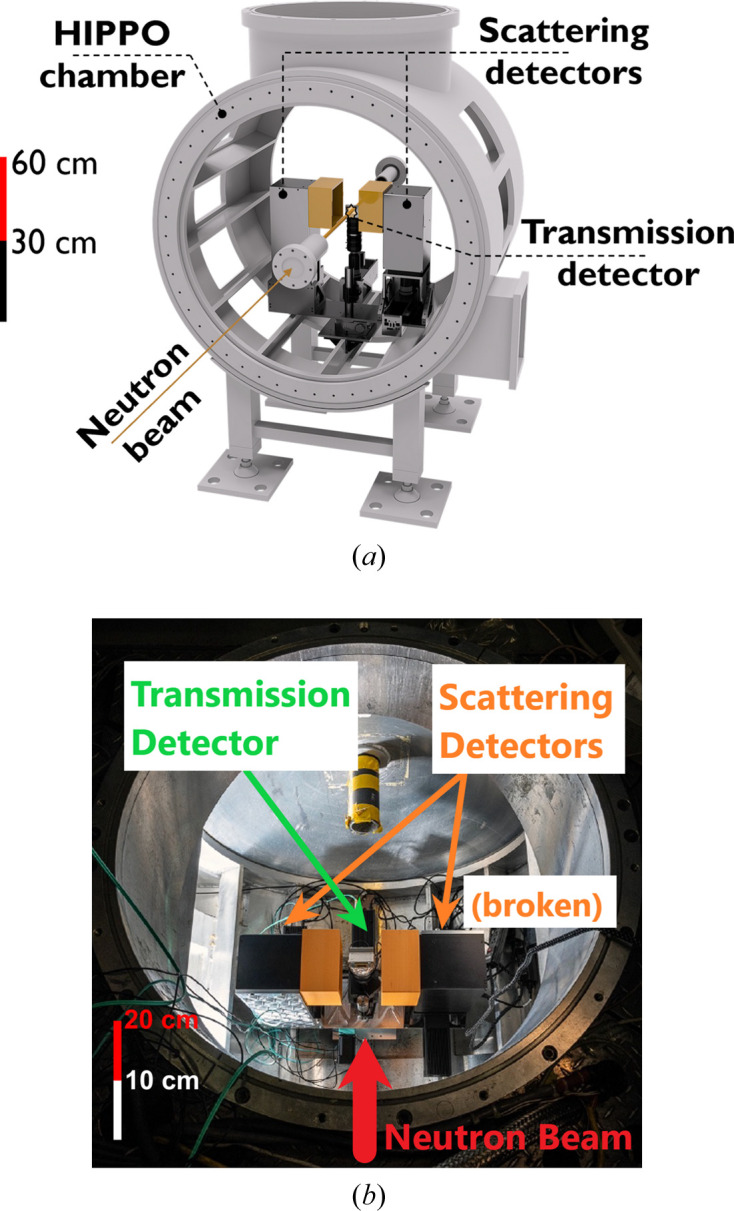
(*a*) Schematic of the experimental setup inside the HIPPO sample chamber with three event-mode cameras (the transmission detector for radiography is much smaller than the large-field-of-view scattering detectors). The camera to the right was removed before the experiment described here because it was broken. (*b*) Photograph of the actual setup from the top of the HIPPO sample chamber (the camera to the right was removed before the experiment described here).

**Figure 2:**
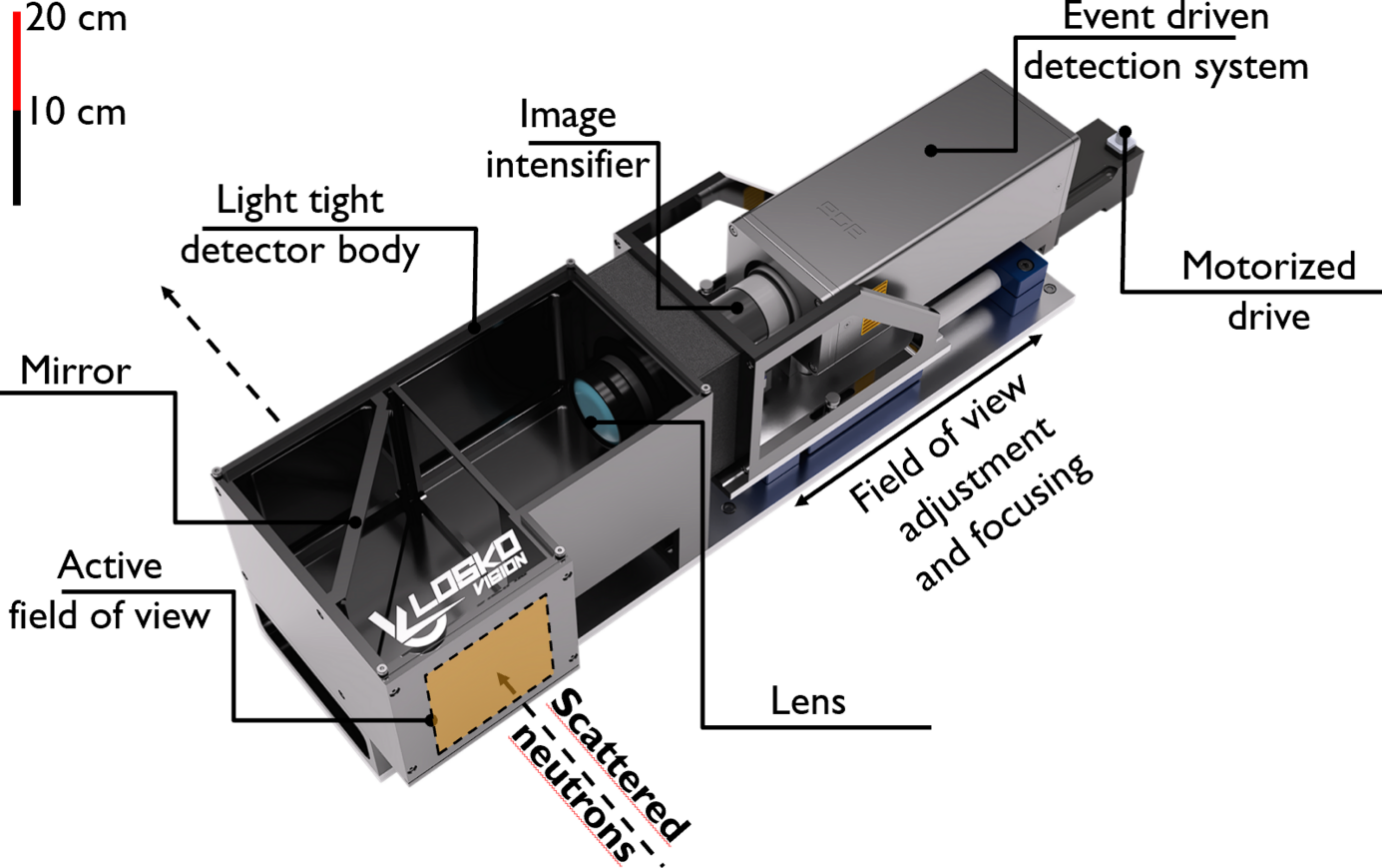
Schematic setup of the camera system used for the diffraction experiment. The active field of view is oriented such that it detects scattered neutrons.

As a first test of imaging-based neutron diffraction analysis, a silicon powder sample in a vanadium can with 9 mm diameter and 50 mm height was measured at the HIPPO beamline. Two event-mode camera systems were deployed for this test (see Fig. 1 ▸): one in the forward transmission direction at about 10 mm distance to the sample for radiography, and one more to the left of the sample (when viewed along the beam direction) at approximately 90° angle to the incident beam and 11.3 cm distance to the sample. Fig. 2 ▸ shows a schematic of the event-mode imaging system employed in this diffraction experiment. The neutron-sensitive area of the detector was a 12 × 12 cm scintillator screen with a 400 µm-thick mixture of silver-doped zinc sulfide (ZnS:Ag) and lithium-6-enriched lithium fluoride [RC Tritec AG, ^6^LiF/ZnS:Ag (ratio 1/2), 450 nm peak emission (Neuwirth *et al.*, 2020 ▸; RC Tritec, 2004 ▸). The scintillator screen was shielded from air scatter neutrons by a ^10^B-containing plastic shielding (orange in Fig. 1 ▸). The light from this scintillator screen was projected on a dual multi-channel plate (in chevron configuration) image intensifier [Photonis, Cricket Hi-QE Green, 5 × 10^5^ gain, P47 phosphor (Exosens Products, 2024 ▸)] via a mirror and an optical lens [Navitar, DO-2595, 25 mm focal length, F/0.95 (Navitar Lenses, 2024 ▸)]. The image intensifier was viewed by a Timepix3-based camera [Amsterdam Scientific Instruments, TPX3Cam (Zhao *et al.*, 2017 ▸; TPX3Cam Product, 2024 ▸)]. All these components were enclosed in a light-tight box.

The scintillator screen of this setup could have a maximum size of 11.5 × 11.5 cm. By changing the optical lens and its distance to the scintillator, scintillator screens larger than 11.5 × 11.5 cm can be utilized, but this requires a larger box. Furthermore, the scintillator material and thickness can also be changed readily as needed for any given experiment. For the diffraction setup described here, a field of view of 15.5 × 15.5 cm was used, thus exceeding the physical size of the scintillator to contain the field of view of the image intensifier. Data from the TPX3Cam were processed following the event centroiding procedures described by Losko *et al.* (2021 ▸). The TPX3Cam has 256 × 256 pixels, achieving an effective resolution of ∼50 µm for the 11.5 × 11.5 cm screen, due to the event centroiding [see below and Losko *et al.* (2021 ▸)].

The maximum event rate of the detector system depends on parameters such as the aperture and the focus of the lens, the image intensifier gain, and the type of scintillator. If these parameters are tuned properly, maximum neutron detection rates of 5 MHz are feasible with ZnS:Ag scintillators. For comparison, the incident flux on the sample for HIPPO is ∼1 × 10^7^ neutrons s^−1^ cm^−2^ at a typical proton current of the linear accelerator of 100 µA (Ino *et al.*, 2004 ▸), providing an upper bound for the total neutron detection rate of the entire detector system in the case that all neutrons are scattered and detected. Assuming isotropically scattered intensity and a scattering detector system consisting of six cameras with the scintillator screens forming a cube around the sample, each camera would have to detect one-sixth of 1 × 10^7^ neutrons s^−1^ = 1.7 × 10^6^ neutrons s^−1^. The maximum detection rate of 5 MHz of the current system is therefore suitable for time-of-flight measurements at HIPPO. The data used in this paper were acquired within 1 h at a nominal proton current of 90 µA, *i.e.* adjusted for fluctuations in the proton current.

### 2.2. Data processing with *GSAS*

The event-mode data were processed following procedures described by Losko *et al.* (2021 ▸). To obtain diffraction spectra, event data from stripes consisting of pixels of five columns (perpendicular to the neutron beam) were integrated and output in *GSAS* data format (Larson & Von Dreele, 2004 ▸). Using a script written in gsaslanguage (Vogel, 2011 ▸), a Rietveld (1969 ▸) time-of-flight to *d*-spacing conversion factor (DIFC in *GSAS*), background parameters and the peak width parameter σ_1_ of the *GSAS* time-of-flight profile function #1 (Von Dreele *et al.*, 1982 ▸) were refined with the silicon lattice parameter held constant at 5.43086 Å. The isotropic atomic displacement parameter *U*_iso_ for the silicon atom was fixed at 0.006719 Å^2^, corresponding to a *B*_iso_ value for room tem­pera­ture reported by Peng *et al.* (1996 ▸), since the limited *d*-spacing range did not allow for a reliable refinement. The incident intensity function as calibrated for the regular 90° HIPPO detector bank was used for the normalization of the diffraction data for the TPX3Cam. The resulting σ_1_ parameter allowed us to find the column with the minimum σ_1_ which is assumed to be at 2ϑ = 90° and these are the data reported here.

### 2.3. Data processing with *MAUD*

Rietveld refinement of the diffraction data with *MAUD* followed the same procedure as described above for the *GSAS* analysis. However, the Rietveld analysis with *MAUD* also comprises the data reduction including calibration of instrument parameters. Reduction of 2D time-of-flight data into histograms analyzable by Rietveld codes can be accomplished with codes such as *MANTID* (Arnold *et al.*, 2014 ▸). However, since for example the reproducibility of the sample alignment cannot be guaranteed, being able to refine calibration parameters such as distance between sample and detector simultaneously with other parameters of the Rietveld refinement has proven crucial in neutron (Wenk *et al.*, 2010 ▸) and synchrotron data analysis (Lutterotti *et al.* 2007 ▸; Wenk *et al.*, 2014 ▸). To implement such an approach for 2D time-of-flight data, *MAUD* was extended to process event-mode data after reduction to location on the scintillator screen and time of flight. Similarly to constant-wavelength 2D data, such as in a synchrotron diffraction experiment, few parameters are required to describe the detector position and orientation. Since neutron data are much more sparse compared with X-ray data, with the additional complication that time-of-flight bins each require sufficient counts for meaningful analysis, the concept of superpixels is introduced in *MAUD*. Superpixels rebin the entire field of view into small segments of *e.g.* 8 × 32 or 1 × 8 pixels, with the short distance being along the axis along the neutron beam (assuming the detector is aligned with the scintillator plane approximately parallel to the incident neutron beam, as is the case here) or the greatest change in 2ϑ, while the superpixel’s long axis is along the azimuthal direction, *i.e.* approximately circumferential to the Debye–Scherrer cones. This resembles the arrangement of ^3^He detector tubes in neutron time-of-flight diffractometers. After defining the superpixel dimensions and starting values for the detector geometry (see below), all events in an event-mode data file are processed and binned in time-of-flight histograms for the superpixels. Using the spatial arrangement of the superpixels within the field of view of the detector and the known diffraction pattern of a calibration substance, in our case silicon powder, a few parameters describing the detector geometry such as sample-to-detector distance, center pixel of the detector, tilt and rotation of the detector plane are refined against all histograms of the superpixels. The superpixels can then be integrated into histograms, similarly to the data reduction for ^3^He tubes for a detector panel or ring, for further refinement.

### 2.4. *McStas* simulation

Simulations with the Monte Carlo code *McStas* (version 3.3) were carried out with a sample-to-detector distance *L*_2_ = 11.3 cm as in the experimental setup described above. Furthermore, simulations were run at *L*_2_ = 83 cm, the nominal distance for the 90° ^3^He detector panels (Wenk *et al.*, 2003 ▸), to validate the reliability of the simulations. *McStas*’ *Source_simple* component was used to generate 8 × 10^10^ neutrons with wavelengths between 0.4 and 3.5 Å emitted from a source area of 2 × 2 cm, which are collimated to a 1.2 × 1.2 cm window at 8.5 m distance, approximately the location of the HIPPO collimation system at the entrance to the sample chamber. The source-to-sample distance of 9 m matches HIPPO’s configuration. After traveling *L*_1_ = 9 m through a vacuum, the neutrons arrive at an air-filled cylinder with *r* = *L*_2_ around a cylindrical silicon powder sample with the same dimensions as in the experiment (9 mm diameter, 50 mm tall). The sample was simulated using the *Union* components *Union_master*, *Union_cylinder* and *Union_make_material* (Bertelsen, 2017 ▸, 2022 ▸) and *NCrystal* (Cai & Kittelmann, 2020 ▸) to create the silicon powder and the surrounding air.

**Figure 3:**
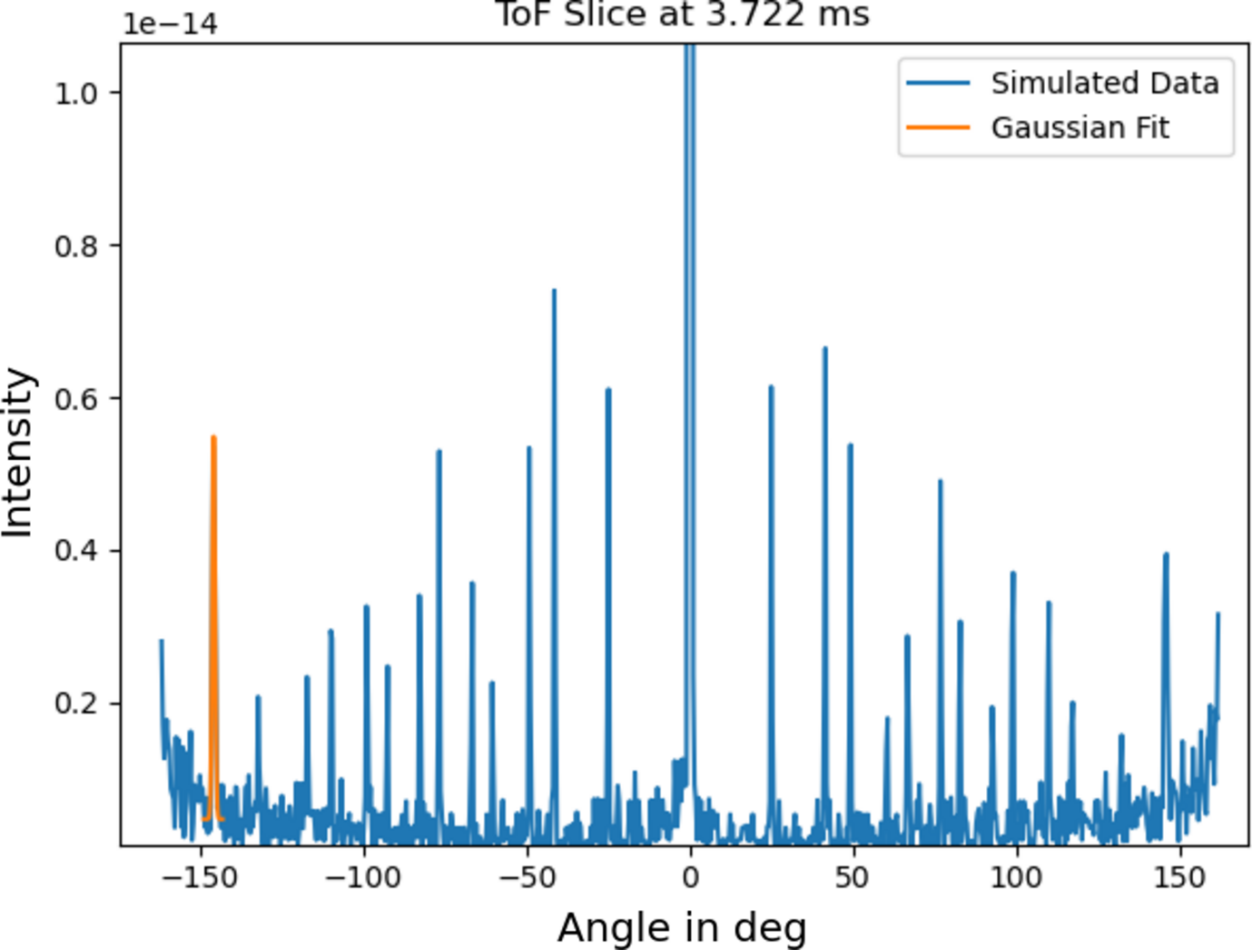
Intensity in arbitrary units versus 2ϑ at an arbitrary TOF of 3.72 ms and *L*_2_ = 83 cm simulated with *McStas* is shown in blue. One of the Gaussian fits used to derive the resolution as a function of 2ϑ is depicted in orange.

Fig. 3 ▸ shows an example of the simulated intensity versus scattering angle at 3.72 ms time-of-flight (TOF), corresponding to an arbitrary *d* spacing of approximately 1 Å, for an *L*_2_ of 83 cm, measured with the *Cyl_monitor_TOF* component. To ensure the detector resolution is not limiting the simulated *d*-spacing resolution, *Cyl_monitor_TOF* was set to nr = 800 radial bins, a height of 4 mm and nt = 1000 temporal bins ranging from tmin = 3.72 ms to tmax = 8.69 ms in the case of *L*_2_ = 83 cm and tmin = 1.00 ms to tmax = 8.06 ms in the case of *L*_2_ = 11.3 cm. To determine the resolution achievable at this distance, all diffraction peaks were fitted with a Gaussian as depicted in Fig. 3 ▸. Assuming the uncertainty in scattering angle 2ϑ as 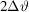 = FWHM = 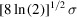 of the Gaussian, the *d*-spacing resolution is calculated as
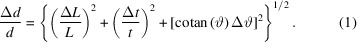
Δ*L* is the uncertainty of the flight path length resulting from the neutron moderation process, scattering from different points in the sample and beam divergence, assumed to be 2.5 cm. The total flight path of the neutrons is *L* = 900 cm + 83 cm = 983 cm or *L* = 900 cm + 11.3 cm = 911.3 cm. Δ*t* is associated with different moderation times creating uncertainty in the neutron’s TOF. Russell *et al.* (1985 ▸) report a neutron pulse width of ∼20 µs for neutron energies between 1 and 50 meV, corresponding to neutron wavelengths from 1.3 to 9 Å and for neutron detectors at 90° a *d*-spacing range from 0.9 to 6 Å. This matches our *d*-spacing range of interest and we therefore assumed Δ*t* = 20 µs for our simulation.

## 3. Results and discussion

**Figure 4:**
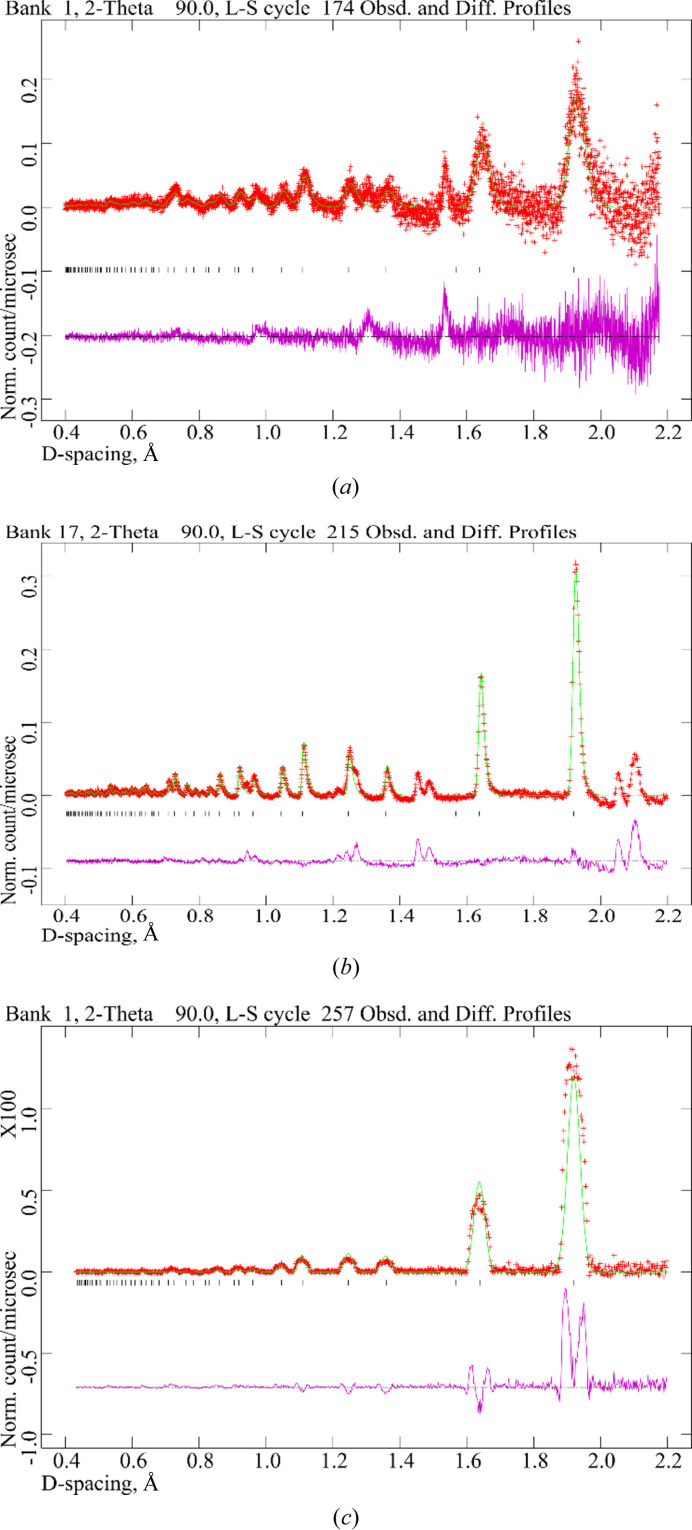
Background-subtracted neutron counts per µs versus *d* spacing at 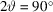 scattering angle from a ∅ = 9 mm Si powder sample: (*a*) measured in 1 h with only five (out of 256) pixel columns of the Timepix3 imaging detector, (*b*) measured in 45 min with 24 ^3^He tubes in HIPPO’s bank 17, and (*c*) simulated with the Monte Carlo code *McStas*. The data are depicted in red, with the *GSAS* fit in green, and the difference between fit and data is shown in purple below. Tick marks indicate calculated peak positions for silicon; the additional peaks observed in (*b*) and Fig. 6 ▸ result from the incident beam diffracting off an aluminium component in the imaging detector, which is to be eliminated in future designs.

Fig. 4 ▸(*a*) shows the background-corrected intensity measured with only five pixel columns of the imaging detector setup (in red) versus *d* spacing at 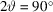 and *L*_2_ = 11.3 cm, including the *GSAS* Rietveld fit in green. As for the ^3^He tubes too, the background was determined by fitting it with *GSAS*. Comparing the results with 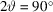 data measured simultaneously with a single ^3^He detector panel consisting of 24 ^3^He tubes at *L*_2_ = 83 cm, depicted in Fig. 4 ▸(*b*), and the *McStas* simulation carried out for *L*_2_ = 11.3 cm, shown in Fig. 4 ▸(*c*), indicates good agreement of the positions of the 422, 331, 400, 311 and 220 silicon diffraction peaks at 1.108, 1.246, 1.358, 1.637 and 1.920 Å, respectively. Because, for a medium-resolution diffractometer such as HIPPO, the constant and quadratic terms σ_0_ and σ_2_ for the peak broadening are zero in practice, the expression for the *d*-spacing-dependent width for the TOF profile function #1 in *GSAS* reduces to σ = σ_1_*d* (Larson & Von Dreele, 2004 ▸). Using the relationship *t* = DIFC × *d* between TOF *t* and *d* spacing *d* yields equation (2) for the Δ*d*/*d* resolution:
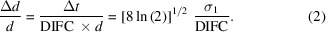


Inserting σ_1_ and DIFC refined with *GSAS*, the imaging detector’s *d*-spacing resolution was determined to be Δ*d*/*d* = 2.1%, which is in good agreement with the *GSAS* analysis of the simulation results (at 90° for *L*_2_ = 11.3 cm) yielding 2.2% resolution. The resolution obtained with the imaging detector system is far from what the detector’s spatial resolution of 50 µm and its temporal resolution of 1.6 ns would allow, which is consistent with the simulation result that the resolution at such distances is mainly determined by sample broadening due to the proximity of the detector to the sample. This means that even larger scintillators can easily be viewed with one camera since the spatial resolution of the camera is not the limiting factor. The significant deviations between simulation data and the Rietveld model for the *McStas* simulation are due to our rather simple model intended to study the effect of instrument and sample geometry (distances, angles *etc*.) on the broadening, not to predict detailed line profiles such as asymmetric broadening caused by the pulsed source *etc*.

To compare the number of diffracted neutrons detected by each detector system, we calculate the solid angles of each. The diffraction data from the TPX3Cam [Fig. 4 ▸(*a*)] were obtained with an active area of 3.48 cm^2^ (5 × 190 out of 256 × 256 pixels viewing 15.5 × 15.5 cm at a distance of 11.3 cm) covering 0.027 sr, whereas the 24 ^3^He (1/2 inch diameter, 12 inches tall, ∼83 cm from sample) tubes of bank 17 [Fig. 4 ▸(*b*)] cover 929.03 cm^2^ or 0.135 sr. To minimize the influence of the higher background detected by the Timepix3 setup, caused by its proximity to the sample, the incident neutron beam and the second imaging detector, only the events in the 1.920 Å diffraction peak were integrated for comparison (after background subtraction). In 60 min of beam at a nominal proton current of 90 µA the Timepix3 detector recorded 1.536 × 10^3^ events, while the ^3^He tubes recorded 1.029 × 10^4^ events in 45 min at the same nominal current. After normalization by solid angle and count time, the imaging detector collected 3.16 events sr^−1^ s^−1^ and the ^3^He tubes detected 5.65 events sr^−1^ s^−1^. Hence, the imaging detector recorded 44.1% fewer events per steradian and second than the ^3^He detector system of the HIPPO instrument. In part, this can be explained by the event-mode readout software for the imaging detector, which caused ∼20% dead time in the version used for this experiment. Ongoing code optimizations will mitigate this in future versions. Furthermore, the scintillator used in this experiment was a standard radiography scintillator not optimized for this diffraction experiment. Gamma rejection using the Timepix3 system is achieved in the software during the photon event processing utilizing the different event shapes of neutron and gamma events (Sykora *et al.*, 2012 ▸; Johnson & Losko, 2023 ▸). The parameters for the gamma rejection depend on scintillator properties and detector configuration, and their optimization for a given experiment is an ongoing development. In the present configuration gamma events are negligible due to the low interaction probability of gammas with the silicon sample. Our observation from preliminary data analysis of TPX3Cam transmission data on HIPPO and the adjacent flight path 5 (FP5), viewing the direct beam including its gamma component, is that compared with previously used detector technology (Tremsin *et al.*, 2017 ▸) the background in both the thermal (<0.4 eV) and epithermal (>0.4 eV) energy ranges for Bragg-edge and neutron absorption resonance analysis, respectively, is extremely low with the Timepix3-based detector setup presented here. Since the beamline characteristics, especially for the thermal/epithermal neutron imaging beamline FP5, are identical to previous work, we conclude that the gamma background rejection in the event-mode processing is very promising. We will report these results in detail in the near future. Therefore, a slightly more gamma-sensitive ^6^Li glass scintillator (∼1 mm thickness) could be used to achieve 90.4% neutron absorption at 2.72 Å compared with the 45.1% the 0.4 mm ZnS:Ag/^6^Li used here provides. While a quantitative comparison of the efficiencies will be the subject of future work, including a comparison of different scintillator screens, the overall efficiencies observed for the two detector technologies tested here are comparable. This is especially true when considering that an event-mode system optimized for radiography was used for this demonstration of diffraction data collection, without any optimizations of the setup for diffraction whatsoever.

**Figure 5:**
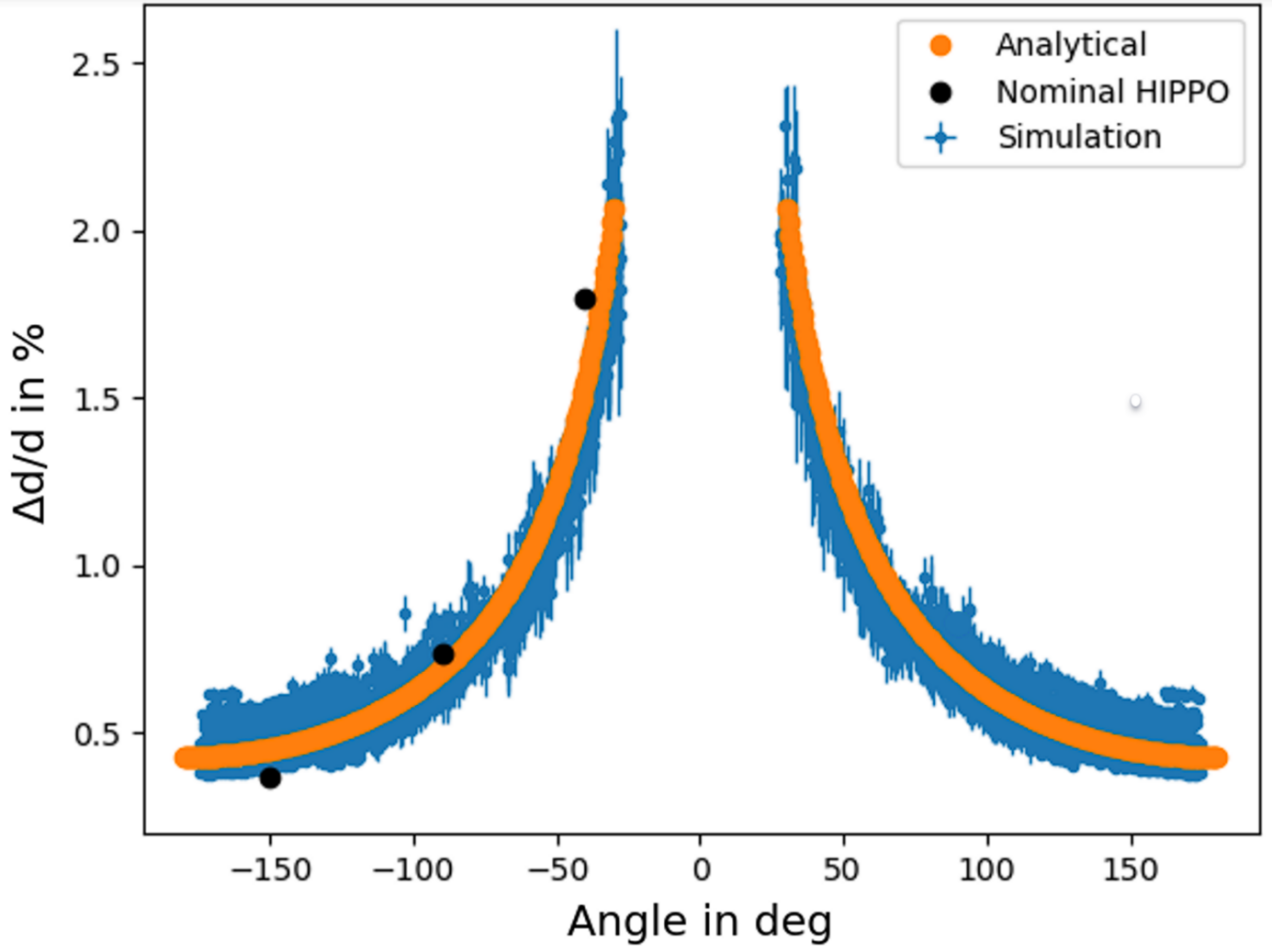
Resolution of a diffraction detector at 83 cm distance to the sample simulated with *McStas* (blue) and nominal data from Wenk *et al.* (2003 ▸) (in black). The orange curve depicts the resolution estimated calculating Δϑ with equation (3). The agreement between simulation and nominal data points validates our *McStas* model, thus allowing us to compare the predicted and observed resolutions for the Timepix3 setup.

To verify the accuracy of resolutions determined with *McStas* as a function of 2ϑ, results are compared with nominal data from Wenk *et al.* (2003 ▸) in Fig. 5 ▸. Simulation and nominal data are in good agreement, validating simulated resolutions. Furthermore, a resolution estimation made using the analytical function 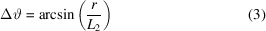
to approximate Δϑ using only *L*_2_ and the sample radius *r* is depicted in orange. The resolution calculated by inserting equation (3) into equation (1) and using the same parameters for *L*, Δ*L*, *t* and Δ*t* as described in Section 2.4, agrees well with nominal and simulated data, illustrating that for approximate resolution assessments time-consuming simulations can be omitted.

**Figure 6:**
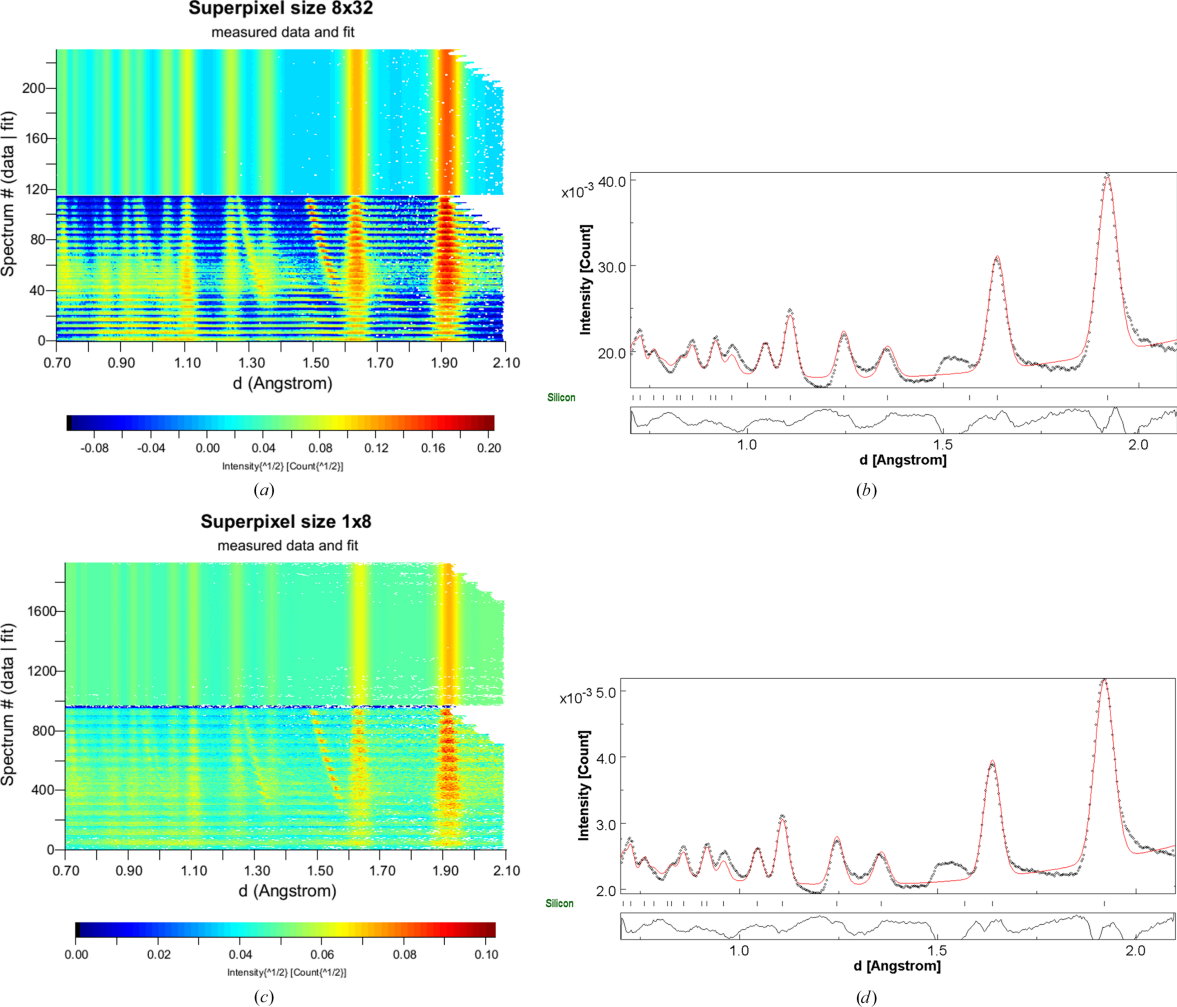
(*a*) Contour plot produced by *MAUD* of diffracted intensity as a function of *d* spacing for 48 superpixels of 8 × 32 size, each shown as horizontal stripes, as raw data (bottom panel) and after the fit (top panel). (*b*) Integrated histogram of all 48 superpixels of 8 × 32 size after the Rietveld fit (red curve) to silicon powder data (black dots) of all individual histograms, with the difference curve below and silicon reflection positions marked by tick marks. (*c*) Contour plot produced by *MAUD* for 2688 superpixels of 1 × 8 size. (*d*) Integrated histogram of all 2688 superpixels of 1 × 8 size. See text for more information.

Fig. 6 ▸ shows the results of the data reduction with *MAUD* for 8 × 32 [(*a*) and (*c*)] and 1 × 8 [(*b*) and (*d*)] superpixel sizes. As for the *GSAS* analysis described above, the silicon lattice parameter and atomic displacement parameter were kept constant during the refinement. Besides three Chebyshev polynomial parameters for the background and the aforementioned detector calibration parameters, only a velocity absorption correction to accommodate the deviations from the estimated incident intensity spectrum and the peak broadening parameters were varied. The raw data (lower panel) in Figs. 6 ▸(*a*) and 6 ▸(*c*) show the presence of spurious diffraction peaks from the aluminium holders of the imaging detector outside the calibrated sample position as diagonal stripes. The much smaller 1 × 8 superpixel size leads to 56 times more superpixels at the cost of reduced counts in each superpixel. However, the resulting peak width in the integrated spectrum in Fig. 6 ▸(*d*) is virtually identical to that obtained with the much coarser 8 × 32 superpixels in Fig. 6 ▸(*b*). This indicates that the geometric resolution of the setup (moderator and sample broadening, short *L*_2_) and not the resolution of the imaging detector is the limiting factor for the Δ*d*/*d* resolution, which is in agreement with the *McStas* findings described above. The main deviations between fit and data in Figs. 6 ▸(*b*) and 6 ▸(*d*) originate from the contributions of the aluminium to the background, which are almost impossible to model and will be mitigated in future versions of this camera by minimizing material contributing to the diffraction signal.

## 4. Conclusion

We demonstrated the first proof-of-concept for an event-mode imaging based scattering detector for neutron scattering data acquisition. This demonstration may pave the way to replacing conventional neutron detectors such as scintillator/photomultiplier or ^3^He detector tubes requiring custom-made electronics and software. The ability to operate imaging, scattering (diffraction, total scattering/pair distribution function analysis, small-angle scattering, reflectometry) and neutron resonance spectroscopy beamlines all with the same data acquisition software and the same simple hardware can greatly reduce investment and operational cost for neutron facilities. The components of a detector system as demonstrated here – TPX3Cam, image intensifier, optical lens, scintillator screen, light-tight box – are all available commercially off the shelf or can be readily fabricated, thus keeping the cost low, especially compared with hardware and software for custom-made readout electronics. While this work demonstrates the feasibility of applying a Timepix3-based system for diffraction detection at least for low- and medium-resolution applications, the applicability to other neutron scattering techniques such as high-resolution single-crystal diffraction, total scattering, small-angle scattering *etc*. with different demands on temperature stability, count rates *etc*. requires further evaluation. Demonstration of the technique with a larger scintillator to cover a larger fraction of 4π is planned for the near future by our team. The detection efficiency of the imaging-based system depends on multiple factors like readout software, image intensifier gain, optical lenses and the scintillator screen, and a detailed analysis of the absolute detection efficiency will be a topic of future publications. However, it is safe to say that detection efficiencies comparable to those of conventional scattering detector technology such as ^3^He tubes can be achieved. The inclusion of an optical lens as well as the ability to design tiled versions of this detector system with multiple TPX3Cams promises scalability to larger detector area coverage with the field of view per camera optimized to handle expected event rates. While scintillator areas up to 625 cm^2^ have been tested successfully in other experiments, we plan to demonstrate scintillator areas exceeding 625 cm^2^ per camera in the near future.

The widely used Rietveld code *MAUD* was adapted to calibrate and process the 2D TOF data generated by the camera-based setup. Building on experience from data analysis for 2D X-ray experiments at synchrotron or X-ray free-electron lase facilities, *MAUD* allows refinement of parameters related to the data reduction, such as sample/detector distances, during the Rietveld analysis rather than separating data reduction and data analysis into two independent steps.

We found that the Δ*d*/*d* resolution obtained with the camera-based setup is as expected for the short distance between sample and detector used in this demonstration: at a sample-to-detector distance of 11.3 cm the resolution from the *GSAS* analysis was 2.1%, in good agreement with simulations carried out with *McStas* yielding Δ*d*/*d* = 2.2% at 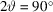. The validity of resolutions determined by *McStas* simulations was established by comparing them with the nominal resolution of HIPPO as published by Wenk *et al.* (2003 ▸). Simulations indicate that at suitable distances, reducing sample broadening, similar resolutions can be achieved with the novel imaging detector system and with the current HIPPO ^3^He-based detector system. Furthermore, more solid angle can be covered by the camera-based system compared with conventional detector panels, thereby detecting more neutrons and reducing measurement time. The camera-based setup will additionally be able to record energy-resolved radiography data of the sample in the transmitted beam direction, therefore enabling simultaneous neutron diffraction and radiography characterization including Bragg-edge (Vogel, 2000 ▸; Woracek *et al.*, 2018 ▸) and neutron absorption resonance imaging (Losko & Vogel, 2022 ▸). Ongoing efforts to further develop this system include the identification of optimal scintillator screens (material and thickness).
